# IL-21 conditions antigen-presenting human γδ T-cells to promote IL-10 expression in naïve and memory CD4^+^ T-cells

**DOI:** 10.1093/discim/kyae008

**Published:** 2024-05-13

**Authors:** Christopher J Tyler, Inva Hoti, Daniel D Griffiths, Simone M Cuff, Robert Andrews, Maximilian Keisker, Raya Ahmed, Hinrich P Hansen, James O Lindsay, Andrew J Stagg, Bernhard Moser, Neil E McCarthy, Matthias Eberl

**Affiliations:** Division of Infection and Immunity, School of Medicine, Cardiff University, Cardiff, UK; Centre for Immunobiology, The Blizard Institute, Barts and The London School of Medicine and Dentistry, Queen Mary University of London, London, UK; Division of Infection and Immunity, School of Medicine, Cardiff University, Cardiff, UK; Division of Infection and Immunity, School of Medicine, Cardiff University, Cardiff, UK; Systems Immunity Research Institute, Cardiff University, Cardiff, UK; Division of Infection and Immunity, School of Medicine, Cardiff University, Cardiff, UK; Division of Infection and Immunity, School of Medicine, Cardiff University, Cardiff, UK; Department of Internal Medicine I, University of Cologne, Cologne, Germany; Centre for Immunobiology, The Blizard Institute, Barts and The London School of Medicine and Dentistry, Queen Mary University of London, London, UK; Department of Gastroenterology, The Royal London Hospital, Barts Health NHS Trust, London, UK; Centre for Immunobiology, The Blizard Institute, Barts and The London School of Medicine and Dentistry, Queen Mary University of London, London, UK; Division of Infection and Immunity, School of Medicine, Cardiff University, Cardiff, UK; Systems Immunity Research Institute, Cardiff University, Cardiff, UK; Centre for Immunobiology, The Blizard Institute, Barts and The London School of Medicine and Dentistry, Queen Mary University of London, London, UK; Division of Infection and Immunity, School of Medicine, Cardiff University, Cardiff, UK; Systems Immunity Research Institute, Cardiff University, Cardiff, UK

**Keywords:** unconventional T-cells, CD4^+^ T-cells, RNAseq, mucosal immunity, antigen presentation

## Abstract

Direct interaction between T-cells exerts a major influence on tissue immunity and inflammation across multiple body sites including the human gut, which is highly enriched in ‘unconventional’ lymphocytes such as γδ T-cells. We previously reported that microbial activation of human Vγ9/Vδ2^+^ γδ T-cells in the presence of the mucosal damage-associated cytokine IL-15 confers the ability to promote epithelial barrier defence, specifically via induction of IL-22 expression in conventional CD4^+^ T-cells. In the current report, we assessed whether other cytokines enriched in the gut milieu also functionally influence microbe-responsive Vγ9/Vδ2 T-cells. When cultured in the presence of IL-21, Vγ9/Vδ2 T-cells acquired the ability to induce expression of the immunoregulatory cytokine IL-10 in both naïve and memory CD4^+^ T-cells, at levels surpassing those induced by monocytes or monocyte-derived DCs. These findings identify an unexpected influence of IL-21 on Vγ9/Vδ2 T-cell modulation of CD4^+^ T-cell responses. Further analyses suggested a possible role for CD30L and/or CD40L reverse signalling in mediating IL-10 induction by IL-21 conditioned Vγ9/Vδ2 T-cells. Our findings indicate that the local microenvironment exerts a profound influence on Vγ9/Vδ2 T-cell responses to microbial challenge, leading to induction of distinct functional profiles among CD4^+^ T-cells that may influence inflammatory events at mucosal surfaces. Targeting these novel pathways may offer therapeutic benefit in disorders such as inflammatory bowel disease.

## Introduction

Control of gut immune responses to the commensal microbiota is essential to maintain healthy barrier function and prevent chronic inflammation. In the rodent intestine, this process is critically dependent on various types of antigen-presenting cells (APCs) and their ability to induce the anti-inflammatory cytokine interleukin (IL)-10 [1]. As a potent suppressor of macrophage and T-cell functions, signalling via IL-10 is crucial for limiting host responses to enteric antigens [2], the absence of which results in chronic inflammation and severe tissue damage [3]. In the transforming growth factor (TGF)-β rich environment of the intestine, IL-10 also plays a major role in stimulating activated B-cells to produce immunoglobulin A (IgA) [4], which regulates gut colonisation by diverse microbes and confers protection against intestinal pathogens [5, 6]. Better understanding of the specific cell types and mechanisms that control IL-10 levels in human blood and tissues will therefore aid the development of novel immunotherapies for both microbial infections and inflammatory diseases.

Classically, professional APC function and control of IL-10 expression in mucosal tissues have been regarded as features restricted to myeloid cells. However, select populations of innate lymphocytes and lymphoid cells can also exhibit APC activity and stimulate conventional T-cell responses [7, 8], and could therefore also be capable of modulating IL-10 expression at epithelial barrier sites. In a murine model, commensal bacteria have been shown to protect against intestinal damage by ‘colonising’ dendritic cells and eliciting expression of IL-10 and the closely related cytokine IL-22 [9]. Subsequent work identified a further requirement for antigen presentation by group 3 innate lymphoid cells (ILC3s) to regulate mucosal IgA production and coating of gut bacteria [10], as well as inducing programmed death of commensal-specific T-cells [11]. Different types of unconventional T-cells, including Vγ9/Vδ2 T-cells, invariant natural killer T (iNKT) cells, and mucosal-associated invariant T (MAIT) cells, have also been observed to shape B-cell antibody production in an APC-like fashion, including the induction of IgA [8, 12, 13]. These data strongly suggest that ‘unconventional’ APCs can fulfil roles distinct from those performed by myeloid presenting cells, and that these functions may be particularly relevant to epithelial barrier defence and control of mucosal inflammation [8, 14]. In this context, ILC3s from paediatric patients with inflammatory bowel disease (IBD) display reduced expression of the antigen-presenting molecule MHC II, perhaps indicating that impaired APC function of this lineage contributes to disease pathology [11]. However, ILC identity appears highly plastic in different environments, and there are major distinctions between the innate lymphocyte compartments of mice and higher primates [15]. It therefore remains unclear to what extent non-myeloid APCs can influence IL-10 expression levels in human blood and mucosal tissues.

Colonic T-cells can express MHC II in response to challenge with specific microbes, which appear to play a dominant role in shaping effector phenotypes in the intestine [14, 16]. Of particular interest in this context are human Vγ9/Vδ2^+^ γδ T-cells due to their unique ability to sense the microbial metabolite (*E*)-4-hydroxy-3-methyl-but-2-enyl pyrophosphate (HMB-PP), which is produced by a large proportion of pathogenic and commensal bacteria [17], via a mechanism that involves the butyrophilin family members BTN2A1 and BTN3A1 [18, 19]. While comprising the major γδ T-cell population in human blood, Vγ9/Vδ2 T-cell activation leads to upregulation of the gut homing receptors integrin β7 and C–C chemokine receptor 9 (CCR9), and consequently these cells are found in both healthy and inflamed intestine [20, 21]. Curiously, activated Vγ9/Vδ2 T-cells display features of professional APCs (‘γδ T-APCs’) and are capable of priming both naïve CD4^+^ T-cells and CD8^+^ T-cells [22]. However, most research on γδ T-APCs has focused on their excellent potential to cross-present antigens to CD8^+^ T-cells [23], and much less is known about their role in inducing CD4^+^ T-cell responses.

Our previous research demonstrated that upon activation in the presence of IL-2 or IL-15, microbe-responsive human Vγ9/Vδ2 T-cells acquire potent APC functions and polarise CD4^+^ T-cells towards expression of interferon (IFN)-γ and IL-22, respectively (but not IL-17) [24]. In contrast, γδ T-APCs generated in the presence of the cytokine IL-21 (γδ_IL-21_ T-APCs), which is closely related to IL-2 and IL-15 and overproduced in active IBD [25], induced lower levels of CD4^+^ T-cell proliferation and lower expression of IFN-γ and IL-22 [24]. Rather than giving rise to T helper 1 (Th1) and Th22-like cells, here we report that γδ_IL-21_ T-APCs are instead well-equipped to induce IL-10 responses by naïve and memory CD4^+^ T-cells, in both allogeneic and antigen-specific autologous settings. Levels of IL-10 production by γδ_IL-21_ T-APC primed CD4^+^ T-cells readily surpassed those produced by CD4^+^ T-cells polarised in the presence of other APC types, including both monocytes and monocyte-derived dendritic cells (DCs). These findings identify an unexpected role for IL-21 in modulating CD4^+^ T-cell responses in microbial infections sensed by Vγ9/Vδ2 T-cells.

## Results

### IL-21 stimulated γδ T-cells induce IL-10 production in naïve and memory CD4^+^ T-cells

**Figure 1:**
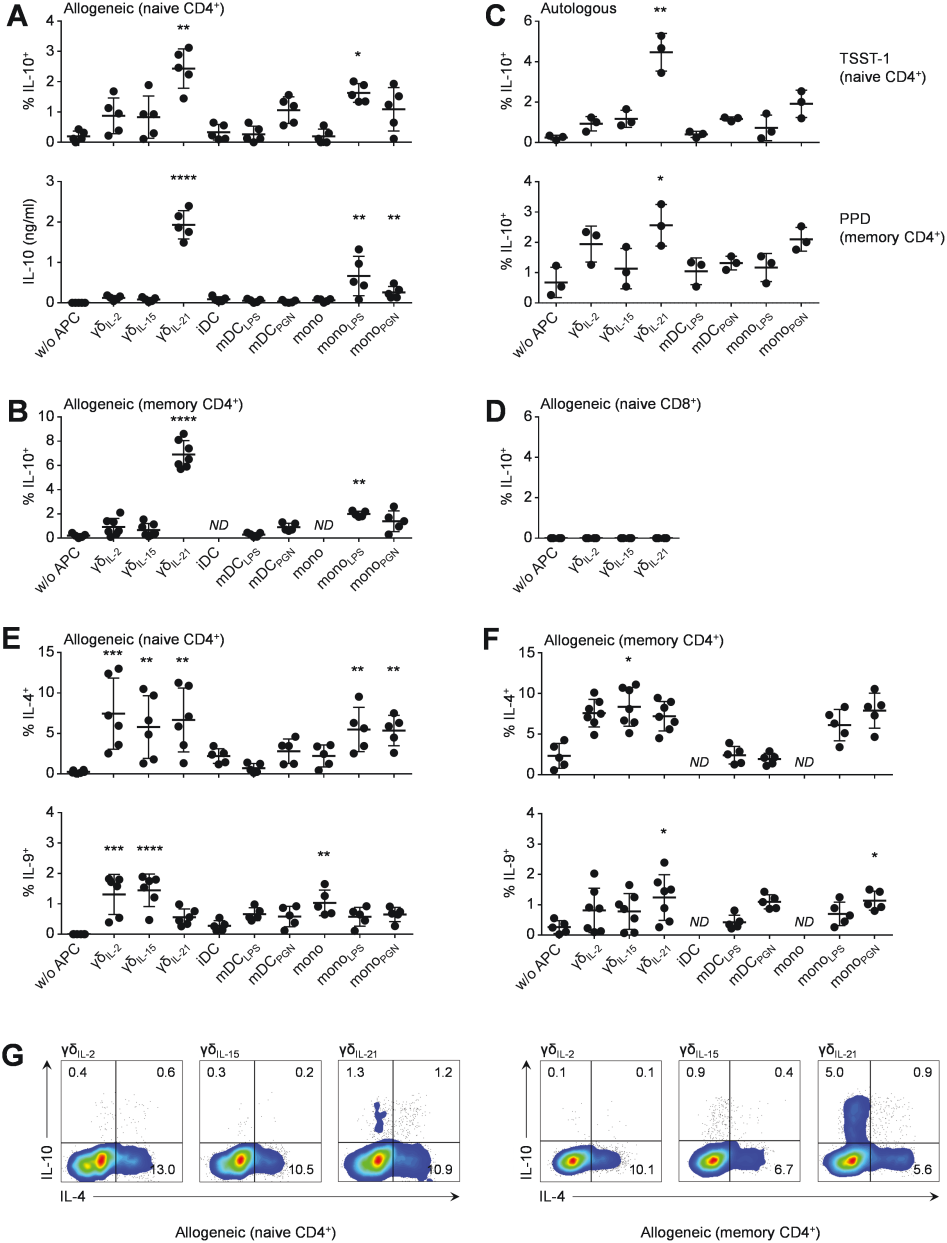
CD4^+^ T-cell polarisation by γδ T-APCs. **(A)** Induction of IL-10 expression in naïve CD4^+^ T-cells by allogeneic γδ T-APCs generated under different conditions, as determined by flow cytometry (top) and ELISA (bottom) upon restimulation after nine days in culture. Myeloid APC controls included immature DCs (iDC), LPS or PGN-matured DCs (mDC_LPS_, mDC_PGN_), freshly isolated monocytes (mono), and LPS or PGN stimulated monocytes (mono_LPS_, mono_PGN_). (**B**) Induction of IL-10 expression in memory CD4^+^ T-cells in response to the allogeneic APC populations shown, as determined by flow cytometry upon restimulation after nine days in culture. (**C**) IL-10 production by superantigen-specific naïve CD4^+^ T-cells in response to autologous APCs pulsed with TSST-1, within the Vβ2^+^ CD4^+^ gate (top), and by microbial antigen-specific memory CD4^+^ T-cells in response to autologous APCs in the presence of PPD (bottom). (**D**) Induction of IL-10 expression in naive CD8^+^ T-cells in response to allogeneic APC populations, determined as above. (**E**) Induction of IL-4 and IL-9 expression in naïve CD4^+^ T-cells in response to allogeneic γδ T-APCs generated under different conditions, as assessed upon restimulation after nine days in culture. The indicated populations of myeloid APCs served as controls. (**F**) Induction of IL-4 and IL-9 expression in memory CD4^+^ T-cells in response to the allogeneic APC populations shown, as determined by flow cytometry upon restimulation after nine days in culture. (**G**) Intracellular staining of IL-10 and IL-4 in naive (left) and memory (right) CD4^+^ T-cells in response to allogeneic γδ T-APCs generated under different conditions. Data in A–C, E, and F were analysed using Kruskal–Wallis tests combined with Dunn’s multiple comparisons tests compared to w/o APC controls (*n* = 5–7); data in *D* were analysed using a Friedman test combined with Dunn’s multiple comparisons tests compared to w/o APC controls (*n* = 4). FACS plots are representative of experiments using cells from four different donors. *ND*, not done

Our previous study showed that unlike γδ T-APCs generated in the presence of IL-2 or IL-15, γδ_IL-21_ T-APCs were poor inducers of IFN-γ and IL-22 expression in naïve CD4^+^ T-cells [24]. This observation, together with the failure to generate any Th17 cells in such assays, prompted us to examine possible alternative outcomes of γδ_IL-21_ T-APC primed CD4^+^ T-cell responses in more detail. Unexpectedly, these experiments demonstrated that γδ_IL-21_ T-APCs were well equipped to induce IL-10 in naïve CD4^+^ T-cells. In mixed lymphocyte reactions with allogeneic responder cells, the ability of γδ_IL-21_ T-APCs to give rise to IL-10^+^ CD4^+^ T-cells was far greater than that of any other γδ T-APC type tested, and surpassed that of lipopolysaccharide (LPS) or peptidoglycan (PGN) activated monocytes or monocyte-derived DCs (Fig. 1A). The unique capacity of γδ_IL-21_ T-APCs to induce IL-10 was particularly apparent by ELISA, showing that γδ_IL-21_ T-APC polarised CD4^+^ T-cells secreted substantial amounts of IL-10 into the culture medium, whereas CD4^+^ T-cells polarised in the presence of other γδ or myeloid APCs did not (with the exception of LPS treated monocytes) (Fig. 1A).

Of note, the capacity of γδ_IL-21_ T-APCs to trigger IL-10 responses was not confined to newly primed naïve CD4^+^ T-cells. Using allogeneic memory CD4^+^ T-cells as responders, γδ_IL-21_ T-APCs readily induced far greater levels of IL-10 than other γδ T-cells, monocytes, or DCs, yielding up to 9% IL-10^+^ cells among memory CD4^+^ T-cells (Fig. 1B). Similar results were obtained in an autologous setting, using *Staphylococcus aureus* toxic shock syndrome toxin-1 (TSST-1) superantigen, which specifically acts on Vβ2^+^ CD4^+^ T-cells, or with *Mycobacterium tuberculosis* purified protein derivative (PPD), which is believed to require antigen uptake and processing (Fig. 1C). These experiments identified a distinct ability of γδ_IL-21_ T-APCs to induce IL-10 responses in both autologous TSST-1 restricted naïve CD4^+^ T-cells, and to a lesser extent, in PPD specific memory CD4^+^ T-cells. Strikingly, γδ_IL-21_ T-APCs failed to induce IL-10 in CD8^+^ T-cells (Fig. 1D), indicating a profound difference between γδ T-APC primed CD4^+^ and CD8^+^ T-cell responses. Taken together, these findings demonstrate that γδ_IL-21_ T-APCs are potent inducers of IL-10 in both naïve and memory CD4^+^ T-cells.

### γδ T-cell induced IL-10^+^ CD4^+^ T-cells are not Th2, Treg or Tfh cells

**Figure 2:**
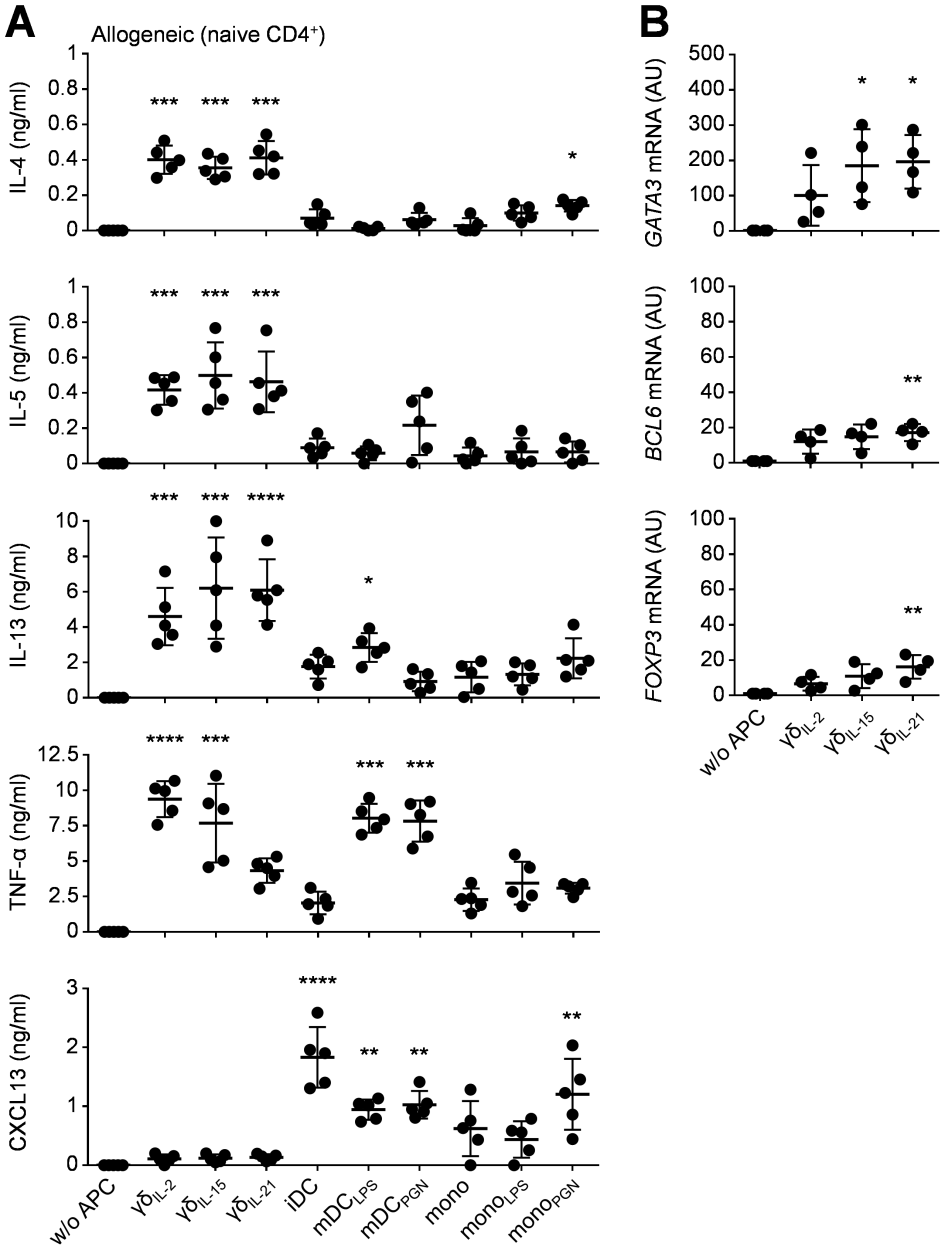
CD4^+^ T-cell polarisation by γδ T-APCs. (**A**) Cytokine secretion by polarised CD4^+^ T-cells generated by co-culture of naïve CD4^+^ T-cells with allogeneic γδ T-APCs or myeloid APCs, as assessed by ELISA upon restimulation after nine days in culture. (**B**) Transcription factor expression by naïve CD4^+^ T-cells after polarisation by γδ T-APCs, as determined upon restimulation after nine days in culture. Expression of transcription factors was determined by RT-PCR after FACS sorting of polarised CD4^+^ T-cells from APC co-cultures. Data in *A* were analysed using Kruskal–Wallis tests combined with Dunn’s multiple comparisons tests compared to w/o APC controls; data in *B* were analysed using a Friedman test combined with Dunn’s multiple comparisons tests compared to w/o APC controls (*n* = 5). AU, artificial units relative to naïve CD4^+^ T-cells

We next attempted to further characterise the γδ T-APC induced population of IL-10^+^ CD4^+^ T-cells. While superior to other γδ T-APC preparations in their ability to induce IL-10, γδ_IL-21_ T-APCs were unable to support preferential induction of IL-4 or IL-9 in naïve (Fig. 1E) or memory CD4^+^ T-cells (Fig. 1F). In fact, the majority of the IL-10^+^ CD4^+^ T-cells induced did not co-express IL-4, irrespective of whether naïve or memory CD4^+^ T-cells were used as responders (Fig. 1G), thereby identifying a distinct population of IL-10^+^ IL-4^−^ CD4^+^ T-cells generated in response to γδ_IL-21_ T-APCs. We were also unable to detect any differences in secretion of the Th2 cytokines IL-4, IL-5, and IL-13 (Fig. 2A) or expression of the Th2 master transcription factor *GATA3* by CD4^+^ T-cells upon stimulation by γδ_IL-2_ T-APC, γδ_IL-15_ T-APCs, or γδ_IL-21_ T-APCs (Fig. 2B).

γδ_IL-21_ T-APCs were only modest inducers of tumour necrosis factor (TNF)-α (Fig. 2A), in agreement with our earlier findings that γδ_IL-21_ T-APCs exhibit only a weak pro-inflammatory profile compared with other γδ T-APC types [24]. γδ_IL-21_ T-APCs also failed to induce relevant levels of C–X–C chemokine ligand 13 (CXCL13), a Tfh signature molecule (Fig. 2A). Finally, CD4^+^ T-cells that had been primed by allogeneic γδ_IL-21_ T-APCs expressed comparatively low levels of *BCL6* and *FOXP3*, the master regulators of Tfh and Treg cells, respectively (Fig. 2B). These findings suggest that γδ_IL-21_ T-APCs give rise to a unique population of IL-10^+^ IL-4^−^ CD4^+^ T-cells that are distinct from Th1, Th2, Th9, Th17, Th22, T follicular helper (Tfh), and T regulatory (Treg) cells. We were unable to determine the extent to which these cells resemble known populations of IL-10 producing type 1 regulatory T (Tr1) or Tr1-like T-cells [26].

### γδ T-cells induce polarisation of IL-10^+^ CD4^+^ T-cells via co-stimulatory interactions through CD80 and CD86

**Figure 3:**
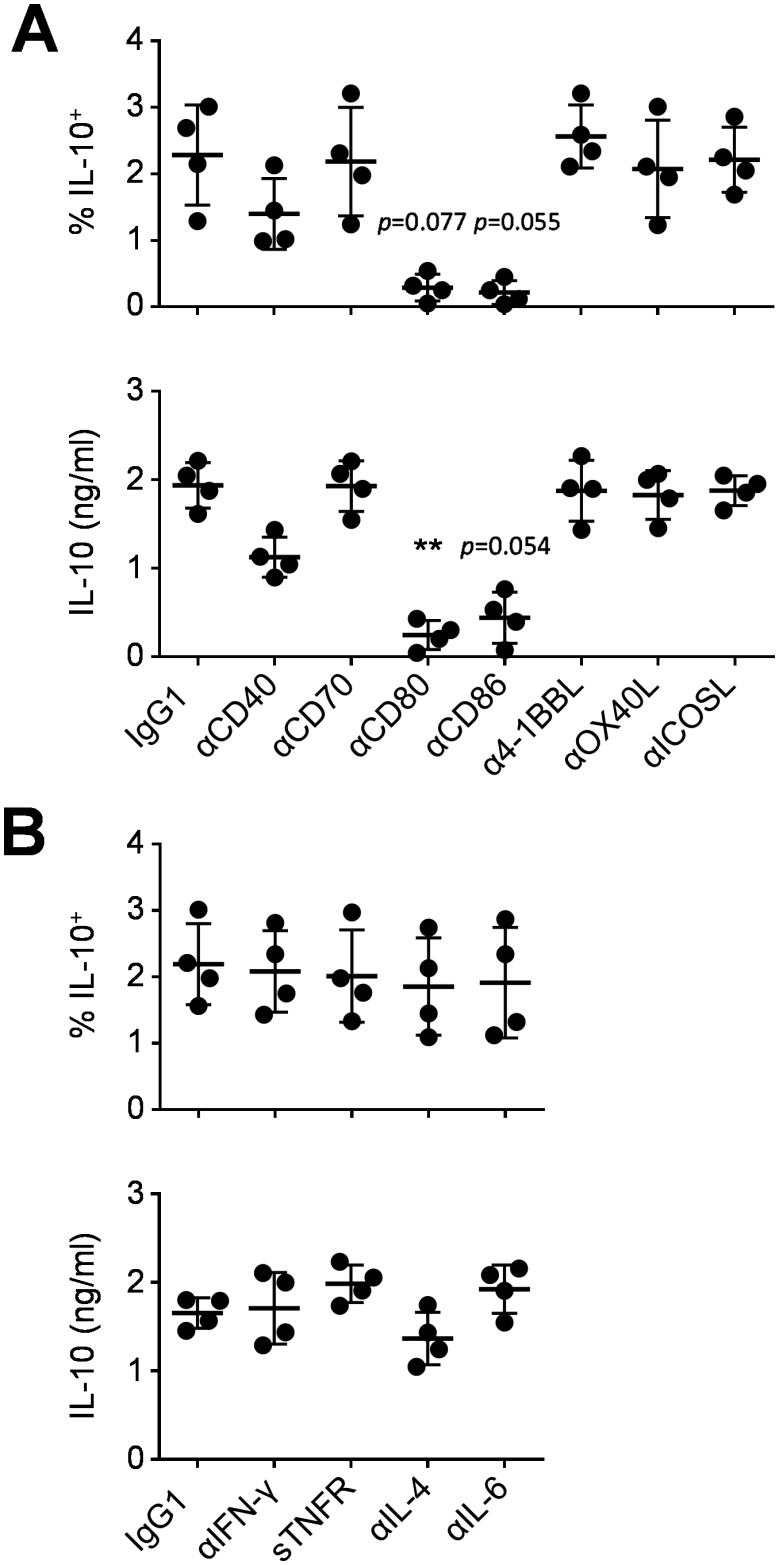
effect of neutralising agents on IL-10 induction in naïve CD4^+^ T-cells. Naïve CD4^+^ T-cells were co-cultured with γδ_IL-21_ T-APCs from allogeneic donors at an APC:responder ratio of 1:10, in the absence or presence of blocking antibodies against co-stimulatory molecules (**A**) or neutralising agents against polarising cytokines (**B**). IL-10 production was determined by flow cytometry and ELISA, upon restimulation after nine days in culture. Data were analysed using Friedman tests combined with Dunn’s multiple comparisons tests compared with IgG1 controls (*n* = 4)

We next sought to identify the IL-10 inducing signals provided by γδ_IL-21_ T-APCs. We previously observed a crucial role for co-stimulation through CD80 or CD86 in supporting proliferation and cytokine production in naïve CD4^+^ T-cells in response to γδ T-APCs [24]. Consistent with these data, blocking either CD80 or CD86 abrogated IL-10 production in co-cultures of naïve CD4^+^ T-cells with γδ_IL-21_ T-APCs (Fig.3A). In contrast, neutralising antibodies against CD70, 4-1BBL, OX40L, or ICOSL had no such effect on the expression of IL-10; inhibition of CD40 signalling exerted a minor influence that failed to reach statistical significance (Fig. 3A). In addition, neutralising the cytokines IFN-γ, TNF-α, IL-4, or IL-6 during the polarisation of naïve CD4^+^ T-cells by γδ_IL-21_ T-APCs did not impair their ability to produce IL-10 (Fig. 3B), in line with our previous observation that γδ_IL-21_ T-APCs did not secret relevant levels of IFN-γ, TNF-α, IL-1β, IL-6, IL-10, IL-12p70, or IL-23 [24]. Thus, the present findings confirmed a role for CD80 and CD86 in the generation of IL-10^+^ CD4^+^ T-cells by γδ_IL-21_ T-APCs but failed to identify additional polarising signals that might be required for the specific induction of IL-10.

### RNAseq analysis of γδ_IL-21_ T-APCs

We next conducted a comprehensive RNAseq analysis of γδ T-APCs generated in the presence of IL-2, IL-15, or IL-21, aiming to define the molecular mechanism underlying selective induction of IL-10 in naïve and memory CD4^+^ T-cells. The closely related γ-chain cytokine IL-7 was included in this analysis as negative control, based on our earlier observation that IL-7 stimulated γδ T-cells do not express APC markers or trigger proliferation of naïve CD4^+^ T-cells [24].

**Figure 4:**
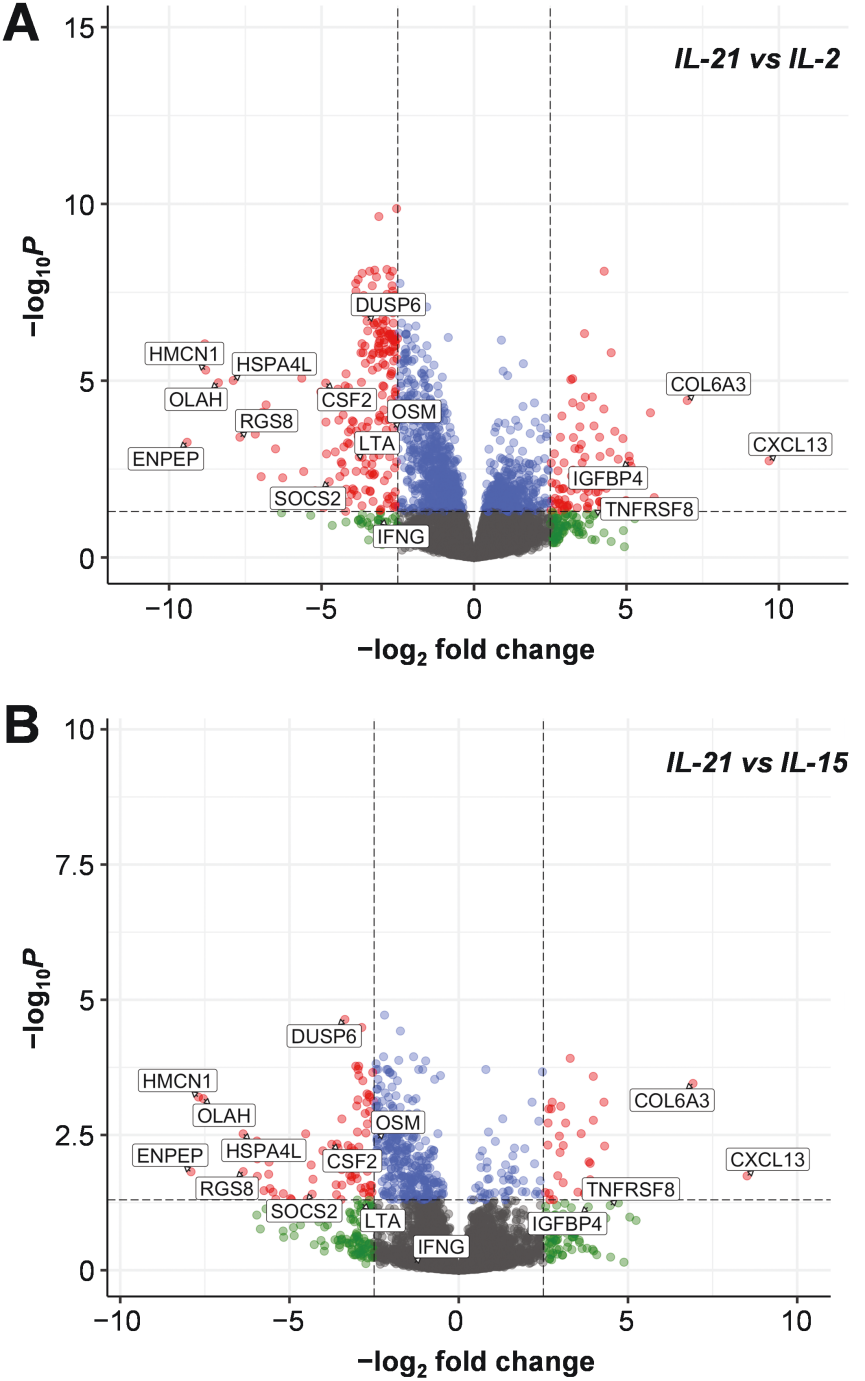
RNAseq analysis of IL-21 stimulated γδ T-APCs. Enhanced volcano plots show differentially expressed genes using γδ T-APCs generated in the presence of IL-2 (**A**) and IL-15 as control **(B)**. Bottom centre in grey, not significant; bottom left and bottom right in green, log_2_ fold change > 2.5; top centre in blue, adjusted *P*-value < 0.05; top left and top right in red, log_2_ fold change > 2.5 and adjusted *P*-value < 0.05. Total variables = 15 460

To identify IL-21 dependent candidates potentially mediating the crosstalk between γδ T-APC and CD4^+^ T-cells, we searched the RNAseq data for genes that were differentially upregulated in the presence of IL-21 but not with IL-2 or IL-15 (Fig. 4). Reassuringly, this RNAseq analysis confirmed a preferential expression of the B-cell attracting chemokine CXCL13 by γδ_IL-21_ T-APCs relative to those generated in the presence of other cytokines, evoking our earlier identification of CXCL13 as signature protein of IL-21 stimulated γδ T-cells [12, 27] (Tables 1 and 2). Besides *CXCL13*, notable transcripts enriched in the presence of IL-21 included *COL6A3* (collagen type VI alpha 3 chain), *IGFBP4* (insulin-like growth factor binding protein 4), *TNFRSF8* (TNF receptor superfamily member 8, also known as CD30), and *TIMP1* (TIMP metalloproteinase inhibitor 1) (Tables 1 and 2). Of note, the RNAseq analysis also confirmed a reduced expression of pro-inflammatory genes by γδ_IL-21_ T-APCs as compared to γδ T-stimulated in the presence of IL-2, such as *CSF2* (GM-CSF), *LTA* (lymphotoxin-α), *IFNG* (IFN-γ), *OSM* (oncostatin M), *CEACAM1* (CD66a), *SOCS2* (suppressor of cytokine signalling 2), and *DUSP6* (dual specificity phosphatase 6) (Table 3), in line with our previous microarray analysis [27].

**Table 1:** top 12 most differentially expressed genes enriched in IL-21 stimulated γδ T-cells compared to IL-2 stimulated γδ T-cells. Only genes with a baseMean value > 50 are shown. lfcSE, standard error of the log2FoldChange; stat, Wald test statistic

GENE_ID	baseMean	log2FoldChange	lfcSE	stat	*P* value	*P* adjusted
*CXCL13*	79.9	9.68	2.41	4.02	5.82 × 10^−05^	0.00186
*COL6A3*	394.7	6.99	1.39	5.03	4.84 × 10^−07^	3.63 × 10^−05^
*IGFBP4*	106.8	5.10	1.27	4.01	6.05 × 10^−05^	0.00191
*JCHAIN*	124.2	4.50	0.79	5.71	1.13 × 10^−08^	1.62 × 10^−06^
*IGHG2*	157.7	4.27	0.61	6.95	3.58 × 10^−12^	8.02 × 10^−09^
*DTX1*	178.6	4.13	1.01	4.11	3.96 × 10^−05^	0.00139
*TNFRSF8*	419.3	3.98	1.45	2.73	0.006249	0.05747
*IGHA1*	155.5	3.88	0.76	5.09	3.65 × 10^−07^	2.89 × 10^−05^
*TIMP1*	2331.2	3.86	1.10	3.51	4.49 × 10^−04^	0.00933
*BCAT1*	1260.2	3.66	1.06	3.44	5.76 × 10^−04^	0.01128
*IGLL5*	53.5	3.65	0.72	5.08	3.71 × 10^−07^	2.92 × 10^−05^
*IGHM*	2306.6	3.64	0.89	4.09	4.24 × 10^−05^	0.00146

**Table 2:** top 12 most differentially expressed genes enriched in IL-21 stimulated γδ T-cells compared to IL-15 stimulated γδ T-cells. Only genes with a baseMean value >50 are shown. lfcSE, standard error of the log2FoldChange; stat, Wald test statistic

GENE_ID	baseMean	log2foldchange	lfcSE	stat	*P* value	*P* adjusted
*CXCL13*	79.9	8.52	2.37	3.60	3.23 × 10^−04^	0.01806
*COL6A3*	394.7	6.92	1.40	4.94	7.82 × 10^−07^	3.52 × 10^−04^
*TNFRSF8*	419.3	4.47	1.46	3.07	0.00214	0.063764
*BCAT1*	1260.2	4.31	1.06	4.05	5.15 × 10^−05^	0.005072
*NAPSB*	397.0	3.98	0.90	4.43	9.33 × 10^−06^	0.001691
*JCHAIN*	124.2	3.98	0.79	5.03	4.92 × 10^−07^	2.61 × 10^−04^
*TIMP1*	2331.2	3.88	1.10	3.53	4.17 × 10^−04^	0.02122
*DTX1*	178.6	3.85	1.01	3.81	1.40 × 10^−04^	0.009911
*IGFBP4*	106.8	3.83	1.26	3.05	0.00231	0.06752
*COL5A3*	1882.7	3.71	1.13	3.29	9.87 × 10^−04^	0.03843
*IGHG2*	157.7	3.29	0.61	5.43	5.70 × 10^−08^	1.21 × 10^−04^
*IGLL5*	53.5	3.16	0.72	4.38	1.18 × 10^−05^	0.00190

**Table 3:** selection of pro-inflammatory signatures genes expressed at lower levels in IL-21 stimulated γδ T-cells compared to IL-2 stimulated γδ T-cells. Only genes with a baseMean value > 50 are shown. lfcSE, standard error of the log2foldchange; stat, Wald test statistic

GENE_ID	BaseMean	log2foldchange	lfcSE	stat	*P* value	*P* adjusted
*CSF2*	5465.1	−4.86	0.92	−5.29	1.25 × 10^−07^	1.16 × 10^−05^
*SOCS2*	1115.3	−4.75	1.32	−3.60	3.16 × 10^−04^	0.00720
*LTA*	22069.8	−3.76	0.92	−4.10	4.17 × 10^−05^	0.00144
*DUSP6*	1042.6	−3.51	0.57	−6.18	6.27 × 10^−10^	2.07 × 10^−07^
*CEACAM1*	61.7	−3.40	1.01	−3.36	7.91 × 10^−04^	0.01379
*IFNG*	34244.7	−3.09	1.22	−2.52	0.01165	0.08594
*OSM*	358.5	−2.66	0.58	−4.60	4.26 × 10^−06^	2.18 × 10^−04^

### Activated γδ T-cells in human blood and intestine express CD30

**Figure 5:**
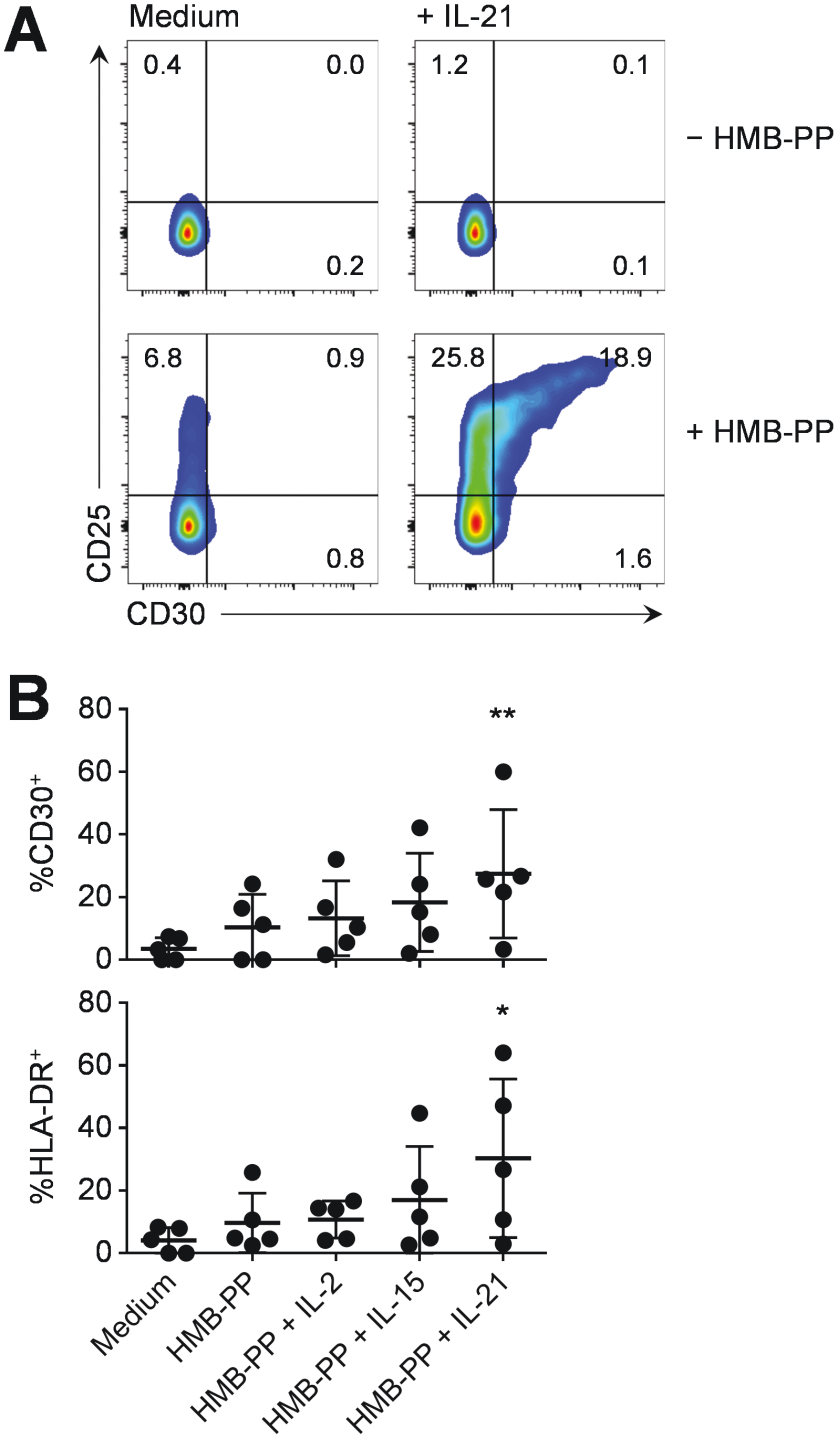
CD30 expression by microbe-responsive γδ T-cells in human blood and gut. (**A**) Human PBMC from a healthy donor were cultured without (top row) or with 10 nM HMB-PP (bottom row) in the absence (left column) or presence (right column) of 20 ng/ml IL-21, and analysed by flow cytometry after three days in culture. Density plots show expression of CD25 and CD30 on single live Vδ2^+^ T-cells. (**B**) Expression of CD30 and HLA-DR on single live Vδ2^+^ T-cells amongst colonic lymphocytes cultured in medium alone or stimulated with 10 nM HMB-PP, either in the absence or presence of 20 ng/ml of the cytokines indicated, and analysed by flow cytometry after three days. Data were generated using colonic biopsies from five different individuals. Data in B were analysed using Friedman tests combined with Dunn’s multiple comparisons tests compared with medium controls (*n* = 5)

During our previous study of autologous CD4^+^ T-cell polarisation by γδ T-APCs, we observed an important role for the TNF superfamily member CD70 in IFN-γ induction, and for the B7 family member ICOSL in IL-22 induction, respectively [24]. Therefore, we postulated that the most promising candidate for a role in polarising IL-10 producing CD4^+^ T-cells was the TNF superfamily member CD30, a relatively poorly characterised co-stimulatory molecule related to CD27, CD40, OX40 (CD134) and 4-1BB (CD137), which is known to interact with CD30L (CD153 or TNFSF8). Flow cytometric analyses showed that resting Vγ9/Vδ2 T-cells from healthy blood were largely negative for CD30 (Fig. 5A). Exposure of PBMC to HMB-PP for 3 days had no discernible effect on CD30 expression, but addition of IL-21 to the medium led to upregulation of CD30 on Vγ9/Vδ2 T-cells, especially on highly activated CD25^hi^ cells (Fig. 5A). As a control, CD4^+^ T-cells in the same PBMC cultures showed only background responses to HMB-PP and IL-21 (data not shown). To the best of our knowledge, these experiments are the first to identify CD30 as a novel activation marker for human γδ T-cells.

Given that IL-10 and IL-21 both play important roles in the gut mucosa, we next investigated expression profiles of CD30 in human colon. These experiments showed only low levels of CD30 and HLA-DR expression by resting Vγ9/Vδ2 T-cells in human gut tissue. Cultures treated with HMB-PP alone showed marginal increases in the expression of both CD30 and HLA-DR but addition of IL-21 had the strongest influence, and was superior to addition of IL-2 or IL-15 (Fig. 5B). These experiments confirmed that CD30 is not only an activation marker for γδ T-cells in human blood but also in the intestine, and that IL-21 in particular is a potent stimulus of CD30 and HLA-DR expression on γδ T-cells in human colon.

**Figure 6:**
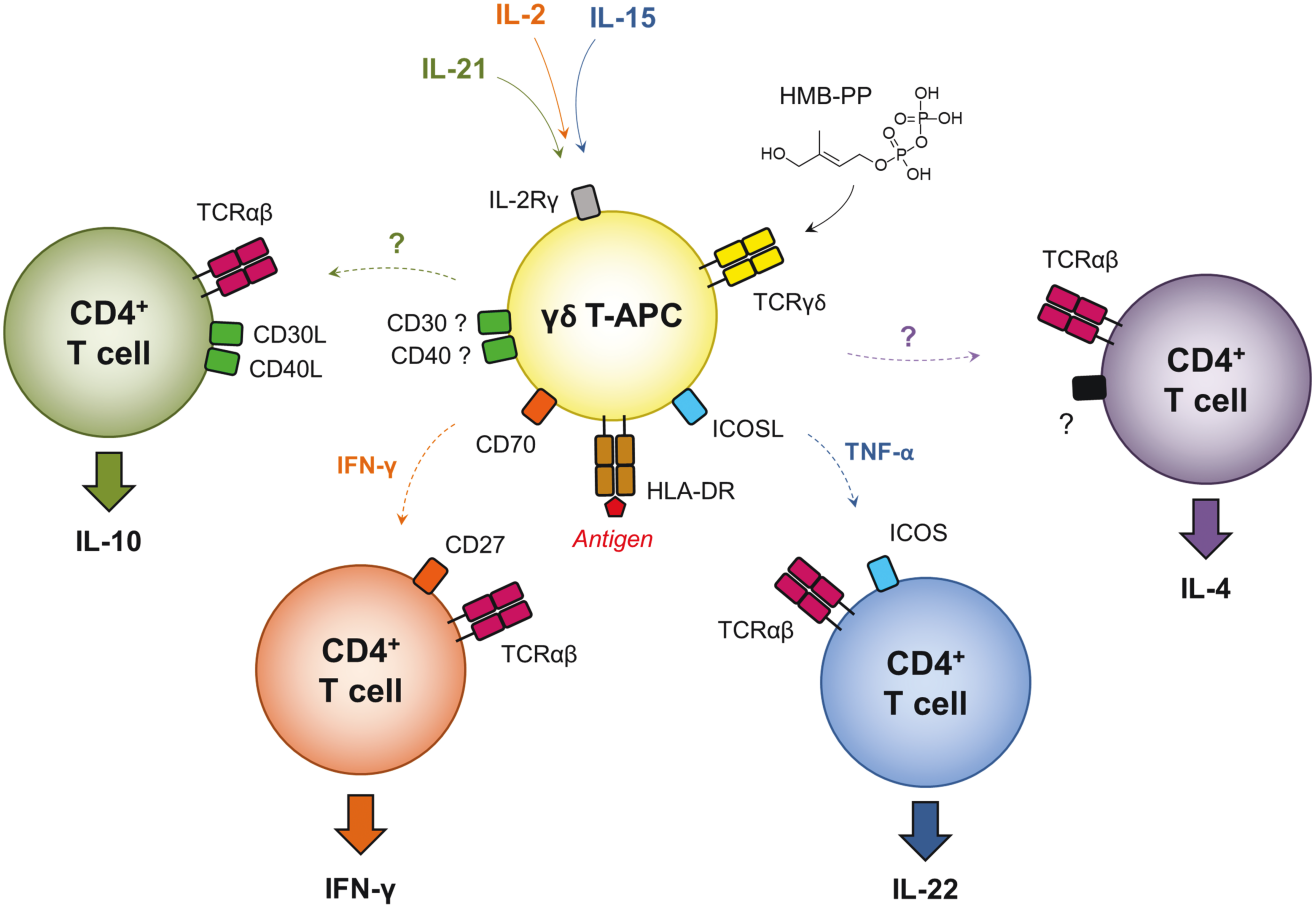
schematic overview of the proposed mechanisms underlying CD4^+^ T-cell polarisation toward distinct effector functions by γδ T-APCs. The microbial metabolite HMB-PP is sensed by Vγ9/Vδ2 T-cells via a process that involves the T-cell receptor (TCR) as well as the butyrophilin family members BTN2A1 and BTN3A1 (not shown). Activated microbe-responsive Vγ9/Vδ2 T-cells express HLA-DR (‘signal 1’) and co-stimulatory molecules such as CD80 and CD86 (‘signal 2’), and have the capacity to induce differentiation of naïve CD4^+^ T-cells towards distinct effector functions (‘signal 3’). Cytokines signalling via the common gamma chain IL-2Rγ (CD132) present during the generation of γδ T-APCs play a pivotal role in this process. Our earlier work defined a role for signalling via IFN-γ and CD27 in the polarisation of IFN-γ producing Th1 cells by γδ_IL-2_ T-APCs, and signalling via TNF-α and ICOS in the polarisation of IL-22 producing Th22 cells by γδ_IL-15_ T-APCs [ref [24]. The present study describes the capacity of γδ_IL-21_ T-APCs to induce IL-10 producing CD4^+^ T-cells, and proposes a role for CD30L and/or CD40L reverse signalling in mediating this effect. Our unpublished data also suggest a potential for other types of γδ T-APCs to polarise naïve CD4^+^ T-cells towards IL-4 producing Th2-like cells (indicated by the question marks), although the underlying mechanism remains to be defined. For the sake of simplicity, interactions via CD80 and/or CD86 that are believed to play a co-stimulatory role in each polarising condition are not shown in this model

Taken together, our data support a model in which activated human Vγ9/Vδ2 T-cells have marked potential to prime naïve CD4^+^ T-cells and, depending on the local cytokine milieu, can drive their differentiation into distinct functional subsets—including Th1 and Th22 cells as published earlier, or into IL-10 producing CD4^+^ T-cells as observed in the present study (Fig.6). Unfortunately, due to lack of commercially available reagents and funding resources we were unable to conduct further studies into the potential role of CD30 in regulating IL-10 responses by CD4^+^ T-cells. Preliminary experiments using a soluble CD30-Fc construct [28] to trigger CD30L reverse signalling in CD4^+^ T-cells, and using anti-CD30 blocking antibodies as control, were inconsistent (data not shown).

## Discussion

IL-10 is an essential regulator of immune activity throughout the human body but plays a particularly pronounced role in the intestine where host leukocytes must co-exist in close association with a rich microbial community. Our new findings show that microbe-responsive γδ T-cells can function as novel APCs capable of priming IL-10 expression in both naïve and memory subsets of conventional CD4^+^ T-cells. Combined with our previous study, which identified a potent ability of these cells to drive colonic T-cell production of the barrier defence cytokine IL-22 [24], our research suggests that human γδ T-APCs may help restrict microbial penetration of the gut epithelium and/or restrain host responses to actively invading pathogens, depending on the prevailing cytokine environment.

IL-10 gene-deficient mice exhibit normal lymphocyte development but suffer from chronic enterocolitis owing to their inability to restrain immune responses to gut microbes [2]. Human patients with mutations in the genes encoding IL-10 receptor subunits are similarly known to develop early onset colitis, which can effectively be ‘cured’ via allogeneic haematopoietic stem cell transplantation to restore normal functioning of this regulatory circuit [3, 29]. However, while a critical role for IL-10 in controlling immune activity has long been established, the cellular sources and specific targets of this cytokine in humans are less well defined. Indeed, rather than being essential producers of IL-10, myeloid lineage cells are instead a critical target for IL-10 signalling in preventing inflammatory disease [30, 31]. IL-10 signalling in macrophages but not T-cells has also been reported to induce regulatory phenotypes that are required for the therapeutic response to anti-TNF treatment in murine colitis [32]. Other major therapies for IBD such as vedolizumab antibody blockade of the gut-homing integrin heterodimer α_4_β_7_ have been observed to impact innate immune cells more potently than their presumed CD4^+^ T-cell targets, in direct correlation with clinical efficacy [33]. It is clear therefore that a more complete understanding of regulatory mechanisms in the human gut is required, in particular an appreciation of the potential for unconventional and innate lymphocyte populations to play important roles.

Our RNAseq analysis identified a range of candidates that were preferentially expressed by γδ T-cells when activated in the presence of IL-21. Out of these, CD30 (TNFRSF8) appeared to be the most promising factor potentially involved in the induction of IL-10 in CD4^+^ T-cells, given the modulatory role of CD30 in other immunological scenarios. In mice, CD30:CD30L signalling has been identified as a critical regulator of Th17 differentiation via effects on T:T interactions and expression of IL-2 [34]. The CD30:CD30L axis has also been implicated in the activation and maintenance of IL-17A-producing γδ T-cells within the mouse gut [35] and in interactions between γδ T-cells and immature B-cells in the mouse spleen [36]. In the absence of this signalling pathway, mice display reduced numbers of γδ T-cells in the mucosa and increased susceptibility to infection with *Listeria monocytogenes,* whereas administration of agonistic anti-CD30 antibody is sufficient to correct these defects [37]. These data suggest that CD30:CD30L signalling is an important mediator of gut γδ T-cell responses to intestinal microbes and subsequent mucosal inflammation. Accordingly, in a rodent model of disease, CD30L^−/−^ mice appear resistant to chemical colitis due to impaired expression of multiple cytokines including IFN-γ, IL-17A, and IL-10 [37]. Conversely, in a mouse model of psoriasis, CD30:CD30L signalling was instead found to reduce pathology by restraining Th17 responses in skin-resident γδ T-cells [38]. Together, these data indicate that therapeutic targeting of the γδ T-cell compartment of different body sites can exert potent effects on tissue inflammation and susceptibility to microbial ingress. Our hypothesis that the CD30:CD30L axis might be involved in γδ T-cell control of CD4^+^ T-helper responses at barrier sites fits with the body of evidence that key regulators of T–T interactions are likely to include novel targets for immunotherapy in a range of different diseases, particularly those affecting epithelial barriers. In fact, colonic T-cell expression of MHC II can be induced by specific microbes, which appear to exert a dominant influence on effector phenotypes among gut lymphocytes [16].

As with other members of the TNF and TNFR superfamilies, both CD30 and CD30L are capable of initiating intracellular signalling cascades, thereby blurring the distinction between a classical ‘receptor’ and its ‘ligand’. So-called reverse signalling through CD30L upon engaging CD30 has been observed in various biological contexts [39, 40]. Of relevance to the present study, CD30-mediated engagement of CD30L was shown to induce cytokine secretion from monocyte-derived immature DCs and promote their differentiation into mature DCs [28]. Similarly, CD30^+^ T-cells modulate Ig class switching as well as IgG, IgA, and IgE production through CD30L expressed on B-cells [41]. A role for CD30L reverse signalling in T-cell differentiation was demonstrated using a viral homologue vCD30 encoded by the ectromelia (mousepox) virus. In mouse models, vCD30 blocked the generation of IFN-γ producing CD4^+^ and CD8^+^ T-cells *in vitro*, and inhibited Th1-driven responses against mycobacterial antigens, but did not impair Th2 inflammatory responses to helminth antigens *in vivo* [42]. Using knockout models, murine CD30L^−/−^ T-cells were found to be defective in their ability to differentiate into Th17 cells *in vivo* and *in vitro* [34]. It has yet to be determined whether the same mechanisms operate in human CD4^+^ T-cells, and whether IL-10-producing cell fates can be promoted by inhibition of Th1 and Th17 differentiation during priming. The role of CD30L reverse signalling during T-cell differentiation into functional subsets clearly warrants closer attention, in both human and animal models.

These considerations regarding CD30L reverse signalling evoke earlier reports of CD4^+^ T-cell co-stimulation through the action of the related molecule CD40 (TNFRSF5) [43]. Cells expressing CD40 were actually able to trigger allogeneic CD4^+^ T-cell responders to proliferate and favoured secretion of IL-10 [44]. CD40 is readily expressed by γδ T-APCs and is also likely to play a functional role [45]. Although it was not differentially enriched in γδ_IL-21_ T-APCs our present experiments showed a partial, albeit insignificant, inhibition of the IL-10 release by γδ_IL-21_ T-APC primed CD4^+^ T-cells when using anti-CD40 blocking antibodies. We speculate that, similar to CD30L, reverse signalling through CD40L might contribute to polarisation of CD4^+^ T-cells by γδ_IL-21_ T-APCs, pending further validation on a functional level.

Another noteworthy mediator identified in the present RNAseq analysis was IGFBP4, a protein that counteracts the effect of insulin-like growth factor-I (IGF-I). Counterintuitively, IGF-I itself has been shown to stimulate IL-10 production in human CD4^+^ T-cells [46], and the action of IGF-I in driving T-cell polarisation towards a Treg phenotype can be blocked by IGFBP4 [47]. However, there are also reports that (at least in mice) IGF-I signalling enhances the programming of pathogenic Th17 cells while suppressing genes commonly associated with Treg cells, including IL-10 [48]. As such, the IGF:IGFBP axis clearly plays a role in affecting the fate of T-cells and can regulate the balance between IL-10 producing CD4^+^ T-cells and other effector T-cell subsets. How these observations can be reconciled with our own RNAseq analysis remains to be addressed in future work.

Taken together, the molecular pathway underlying this potent ability of γδ_IL-21_ T-APCs to induce IL-10 expression in naïve and memory CD4^+^ T-cells remains largely unresolved, and we invite the scientific community to continue our investigations. Of note, the mechanisms postulated here may also be of relevance in tissues other than the gut. While IL-21 is overproduced in the mucosa of IBD patients, IL-21 is a signature marker of Tfh cells involved in driving the germinal centre reaction in secondary lymphoid tissues. Since both IL-10 and IL-21 induce antibody class switching towards IgA [4, 49], and IL-21 stimulated γδ T-cells promote IgA production by human B-cells [12], it is possible that IL-21 fine-tunes the outcome of γδ T-cell interactions with CD4^+^ T-cells in the mucosa and draining lymph nodes to regulate subsequent humoral responses, in accordance with recent evidence showing that lymphatic migration of tissue-derived unconventional T-cells is a key determinant of site-specific immunity [50]. In this context, it is worth noting that patients with IgA nephropathy who are characterised by an increase in circulatory IgA antibodies with aberrant glycosylation (which form immune complexes in the kidneys that ultimately lead to glomerular damage), show a markedly different γδ T-cell repertoire in the gut compared with healthy controls [51]. Modulation of IL-21 as well as CD30, CD40, and/or IGFBP4 expression may therefore exert profound effects on immune responses in barrier tissues and systemically, hence further efforts to investigate these pathways and their immunotherapeutic potential are clearly warranted. We are hopeful that a joint effort by researchers in the field can help solve this puzzle soon.

## Methods and materials

### Tissue samples

Biopsies of colonic mucosa were obtained from patients undergoing colonoscopy for cancer screening or investigation of rectal bleeding but with no significant findings. Additional mucosal tissue (terminal ileum and colon) was obtained from patients undergoing surgical resection for colorectal cancer or for non-inflammatory intestinal motility disorders. Endoscopic biopsies or equivalent size pieces of resected intestinal tissue were washed in calcium and magnesium-free HBSS containing 1 mM dithiothreitol (Sigma) for 15 min to remove mucus and faeces, followed by incubation in 1 mM EDTA for 1 h under constant shaking to remove the epithelium. Mucosal tissue pieces were then transferred into 24 well plates for organ cultures in complete tissue medium containing 30 U/ml IL-2 and 20 ng/ml IL-15, in the presence or absence of 10 nM HMB-PP. After 3 days, intact tissues were discarded and the egressed leukocytes seeded into 96 well round bottom plates for a further 4 days. At the end of the culture period, intestinal Vγ9/Vδ2 T-cells and CD4^+^ T-cells were each sorted to > 99.2% purity.

### Cell isolation from blood and APC generation

PBMC were isolated from heparinised venous blood of healthy donors or from blood bags supplied by the Welsh Blood Service (Velindre NHS Trust) using Lymphoprep (Axis-Shield). CD14^+^ monocytes (> 99% purity) were purified from PBMC using anti-CD14 microbeads (Miltenyi). Immature DCs (iDCs) were derived from monocytes over 5–6 days in the presence of 50 ng/ml GM-CSF (Miltenyi) and 50 ng/ml IL-4 (Miltenyi). Maturation of iDCs into mature DCs (mDCs) and activation of freshly isolated monocytes were achieved via stimulation for 24 h with 100 ng/ml *Salmonella abortus equi* LPS (Sigma), or 1 µg/ml peptidoglycan (PGN; Sigma). Vγ9^+^ T-cells and Vδ2^+^ T-cells (each > 99% purity) were isolated using anti-TCR-Vγ9:PECy5 (Beckman Coulter) or anti-TCR-Vδ2:PE mAbs (BD Biosciences), combined with anti-PE microbeads (Miltenyi). γδ T-APCs were generated by co-culture of purified blood γδ T-cells with irradiated monocytes (50 Gy) at a 10:1 ratio, in the presence of 10 nM HMB-PP (kindly provided by Hassan Jomaa, Justus-Liebig University Giessen, Germany) with or without 100 U/ml IL-2 (Proleukin; Novartis), 20 ng/ml IL-7 (Peprotech), 20 ng/ml IL-15 (Miltenyi) or 20 ng/ml IL-21 (Zymogenetics). γδ T-APCs were cultured for 3 days and further purified either by positive selection or cell sorting to purities > 99.2%. Short-term γδ T-cell stimulation assays were performed in PBMC cultures over 3 days, using 10 nM HMB-PP with or without addition of cytokines. Bulk CD4^+^ and CD8^+^ T-cells (each > 95% purity) were isolated from PBMC via negative selection using the corresponding CD4^+^ and CD8^+^ T-cell Isolation Kits, respectively (Miltenyi). For isolation of naïve and memory subsets, bulk T-cells were labelled with anti-CD4 or anti-CD8 mAbs, together with anti-CD45RA (all from BD Biosciences) and anti-CCR7 mAbs (Biolegend), and sorted using a FACSAria II (BD Biosciences) to obtain CD45RA^+^ CCR7^+^ naïve and CD45RA^−^ CCR7^−^ memory populations, with > 99.4% purity in each case as confirmed by flow cytometry.

### APC assays

All cultures were performed using RPMI-1640 medium supplemented with 10% foetal calf serum, 50 µg/ml penicillin/streptomycin, 2 mM L-glutamine, 1% sodium pyruvate, and 100 µM non-essential amino acids (Life Technologies). For mixed lymphocyte reactions, APCs and allogeneic CD4^+^ or CD8^+^ T-cells were co-cultured at a 1:10 ratio, as described before [24]. γδ T-APCs were irradiated at 12 Gy prior to co-culture with responder cells. For antigen-restricted responses, APCs were pulsed with 1 ng/ml TSST-1 (Toxin Technology) for 1 h and washed three times prior to co-culture with autologous CD4^+^ T-cells. To assess CD4^+^ T-cell responses to complex antigen preparations, APCs were cultured with 1 μg/ml PPD (Statens Serum Institut, Copenhagen, Denmark) for the final 24 h of APC generation and washed three times prior to co-culture with autologous CD4^+^ T-cells. Co-cultures were incubated for five days to assess proliferation of responder cells labelled with CFSE (Life Technologies), or for nine days prior to analysis of cytokine and transcription factor expression. For blocking of co-stimulatory molecules, APCs were pre-incubated with neutralising mAbs for 2–3 h and washed three times, prior to addition of CD4^+^ T-cells; for inhibition of APC-derived cytokines, appropriate blocking reagents were added directly to MLR assays. Blocking reagents used were anti-ICOSL (9F.8A4; Biolegend), anti-IFN-γ (B27), anti-IL-4 (8D4-8), and anti-IL-6 (MQ2-13A5) from Biolegend; anti-4-1BBL (H41BB-M127), anti-CD80 (2D10.4), and anti-CD86 (IT2.2) from BD Biosciences; anti-OX40L (MAB10541) from R&D Systems; sTNFR p75-IgG1 fusion protein (etanercept/Enbrel; Amgen); and anti-CD30 (HeFi-1) [39]. Agonistic reagents included anti-CD3 (OKT3), anti-CD28 (CD28.2), and anti-ICOS (ISA-3) from eBioscience; soluble CD70 (sCD70) from Jannie Borst, Netherlands Cancer Institute; and sCD30-Fc [39].

### Flow cytometry

Cells were acquired on an eight-colour FACSCanto II (BD Biosciences) and analysed with FlowJo 10.1 (TreeStar). Anti-mouse antibody reactive beads were used to set compensation (Life Technologies). Single live lymphocytes and monocytes were gated based on light scatter and side scatter characteristics, and the exclusion of fixable Aqua dead cell stain (Invitrogen), followed by gating based on fluorescence minus one controls [24]. For surface phenotyping, anti-CD4:APC-H7 (RPA-T4), anti-CD45RA:APC (HI100), anti-TCR-Vδ2:PE (B6.1), and anti-HLA-DR:APC-H7 (L243) were from BD Biosciences; anti-TCR-Vγ9:PE-Cy5 (Immu360) from Beckman Coulter; anti-CD25:APC (BC96) from eBioscience; anti-CD3:BV421 (UCHT-1) and anti-CD14:BV421 (M5E2) from Biolegend; and anti-CD30:FITC (BerH2) from Invitrogen; together with appropriate isotype controls. For detection of intracellular cytokines, cells were restimulated with 10 ng/ml phorbol 12-myristate 13-acetate (Sigma) and 1 µM ionomycin (Sigma) for 5 h, and cultures were supplemented with 10 µg/ml brefeldin A (Sigma) during the last 4 h of the incubation period. Intracellular cytokines were detected using anti-IFN-γ:BV421 (4S.B3), anti-IL-9:AF647 (MH9A4) and anti-IL-10:PE-Cy7 (JES3-9D7) from Biolegend; anti-IL-17A:APC (eBio64DEC17) and anti-IL-22:PE-Cy7 (22URTI) from eBioscience); and anti-IL-4:PE (8D4-8) from BD Biosciences.

### ELISA

Cell-free supernatants from resting or stimulated γδ T-cell, DC or monocyte cultures were collected after 3 days incubation. Supernatants from 50 000 polarised CD4^+^ T-cells were collected after 24 h incubation with 10 ng/ml PMA and 1 µg/ml ionomycin. Supernatants from intestinal tissue cells were collected after 3 days in culture. Soluble cytokines were detected using conventional ELISA kits for IFN-γ (Biolegend); IL-4, IL-5, IL-10, IL-13, IL-17, and IL-22 (eBioscience); and CXCL13 (R&D Systems). All samples were measured in duplicate on a Dynex MRX II reader.

### qPCR analysis of CD4^+^ T-cells

CD4^+^ T-cell responders were sorted from co-cultures with irradiated APCs to purities >99.1%. Total RNA was extracted using the RNeasy Micro Kit (Qiagen), examined qualitatively and quantitatively using a NanoDrop ND1000 (Thermo Fisher Scientific) and used to generate cDNA with the SuperScript VILO cDNA Synthesis Kit (Thermo Fisher Scientific). Transcripts were quantified by real-time quantitative PCR (qPCR) using the ViiA7 Real-Time PCR System (Thermo Fisher Scientific) [24]. Predesigned TaqMan Gene Expression Assays and the Taqman Universal Master Mix II (no UNG) were used according to the manufacturers’ instructions: *TBX21*, Hs00203436_m1; *GATA3*, Hs00231122_m1; *RORC*, Hs01076112_m1; *AHR*, Hs00169233_m1; *FOXP3*, Hs01085834_m1; *BCL6*, Hs00153368_m1; and *PPIL2*, Hs00204962_m1 (all from Thermo Fisher Scientific). All samples were measured in triplicate. Measured mRNA abundance was normalised to *PPIL2* (cyclophilin) using the ExpressionSuite Software (Thermo Fisher Scientific), and is presented as arbitrary units (AU).

### RNAseq analysis of γδ T-cells

Monocytes were freshly isolated and irradiated at 50 Gy prior to co-culture with freshly isolated autologous γδ T-cells at a monocyte to γδ T-cell ratio of 1:10. Cultures were treated with 10 nM HMB-PP alone, or in combination with 100 U/ml IL-2, 20 ng/ml IL-7, 20 ng/ml IL-15, or 20 ng/ml IL-21. After three days in culture, CD3^+^ Vδ2^+^ T-cells were enriched by cell sorting using a FACSAria II (BD Biosciences), to purities > 99.8%. Total RNA was extracted from cell pellets using the RNeasy Micro Kit (Qiagen) and examined qualitatively and quantitatively using the TapeStation 2200 system (Agilent) and a Qubit fluorometer (Thermo Fisher Scientific). Ribosomal RNA was depleted as part of the Illumina Stranded Total RNA Prep protocol using coated magnetic beads; the remaining RNA was fragmented and denatured, and first- and second-strand cDNA synthesis and anchor ligation was carried out according to the manufacturer’s instructions. Resulting libraries were quality-controlled using the TapeStation 2200 and Qubit systems, and pooled in equimolar amounts. Samples were subjected to paired-end sequencing with 75 bp reads on a NextSeq 500 sequencer (Illumina), generating >50 million reads per sample.

### Bioinformatics

RNA sequencing data were processed taking advantage of the Nextflow nf-core RNASeq (version 3.14.0) pipeline [52, 53], using the Hisat2 aligner, and removing ribosomal reads. The Binary Alignment Map files produced from this pipeline were used as input for featureCounts (Subread package, version 2.0.1), to obtain a count table, using ENSEMBL *Homo sapiens* GRCh38 (version 108) as reference genome for alignment and feature counting [54]. The count table was then imported into R version 4.3.1 using RStudio IDE version 2023.6.1.524 (Posit Software). Differential gene expression analysis was carried out using DESeq2 (version 1.42.0), and genes with a log_2_ fold change >2.5 and an adjusted *P*-value < 0.05 were considered differentially expressed. Gene expression was visualised using EnhancedVolcano (version 1.20.0).

### Statistical analysis and data presentation

Statistical analyses were performed using GraphPad Prism 6.0 software. Differences between groups were analysed using Kruskal–Wallis tests combined with Dunn’s post-tests; matched data were analysed using Friedman tests combined with Dunn’s multiple comparisons tests. In the graphs, each data point represents an individual donor; asterisks depict statistically significant differences: ^*^P < 0.05; ^**^P < 0.01; ^***^P < 0.001; ^****^P < 0.0001. Horizontal lines display the mean, error bars indicate the standard deviation.

## Data Availability

The raw RNAseq data underlying this article are available from ArrayExpress under accession number E-MTAB-13875. All other data will be shared upon reasonable request to the corresponding author.

## Abbreviations

APC: antigen presenting cell;
BTN: butyrophilin;
CCR: C–C chemokine receptor;
CFSE: carboxyfluorescein succinimidyl ester;
CXCL: C–X–C chemokine ligand;
DC: dendritic cell;
HLA-DR: human leukocyte antigen DR isotype;
HMB-PP: (*E*)-4-hydroxy-3-methyl-but-2-enyl pyrophosphate;
IBD: inflammatory bowel disease;
iDC: immature dendritic cell;
IFN: interferon;
IgA: immunoglobulin A;
IGFBP4: insulin-like growth factor binding protein 4;
IL: interleukin;
ILC: innate lymphoid cell;
iNKT: invariant natural killer T;
LPS: lipopolysaccharide;
PGN: peptidoglycan;
PBMC: peripheral blood mononuclear cells;
PPD: purified protein derivative;
RNAseq: RNA sequencing;
Tfh: T follicular helper;
TGF-β: transforming growth factor-β;
Th: T helper;
TNF: tumour necrosis factor;
TNFRSF: TNF receptor superfamily member;
TNFSF: TNF superfamily member;
Tr1: type 1 T regulatory;
Treg: T regulatory;
TSST**-1**: toxic shock syndrome toxin-1.
